# First‐Line Lorlatinib in Striatin–Anaplastic Lymphoma Kinase‐Positive Lung Adenocarcinoma: A Case Report and Literature Review

**DOI:** 10.1002/rcr2.70750

**Published:** 2026-09-03

**Authors:** Masaru Maebeya, Shinobu Tamura, Takeshi Shono, Takanobu Hoso, Hitomi Tatsuta

**Affiliations:** ^1^ Internal Medicine III Wakayama Medical University Hospital Wakayama Japan; ^2^ Department of Respiratory Medicine Wakayama Rosai Hospital Wakayama Japan; ^3^ First Department of Internal Medicine Wakayama Medical University Wakayama Japan; ^4^ Department of General Medicine Wakayama Rosai Hospital Wakayama Japan

**Keywords:** brain metastases, lorlatinib, non‐small cell lung cancer, STRN–ALK fusion, tyrosine kinase inhibitor

## Abstract

Striatin–anaplastic lymphoma kinase (STRN–ALK) is a rare fusion variant in non‐small cell lung cancer (NSCLC), and the efficacy of first‐line lorlatinib for this subgroup is poorly defined. We report a 45‐year‐old treatment‐naïve male with STRN‐ALK‐positive lung adenocarcinoma and symptomatic brain metastases. Following stereotactic radiosurgery for intracranial lesions, the patient initiated lorlatinib at 100 mg once daily as the first systemic therapy. After starting treatment, neuropsychiatric side effects necessitated a gradual reduction in dosage to 75 mg and then to 50 mg. The patient achieved a durable partial response with sustained intracranial disease control for over 1 year. Adjusting the dosage improved tolerability without compromising efficacy, allowing for ongoing treatment. This case demonstrates that first‐line lorlatinib can achieve durable systemic and intracranial disease control in STRN–ALK–positive NSCLC, including in patients presenting with brain metastases, while maintaining antitumor efficacy despite dose reduction.

## Graphical abstract

A 45‐year‐old male with STRN–ALK‐positive lung adenocarcinoma and brain metastases achieved a significant and durable response to first‐line lorlatinib. Together with our literature review of 15 previously reported cases, this case supports further evaluation of lorlatinib as a first‐line treatment option for patients harbouring this rare ALK fusion variant.

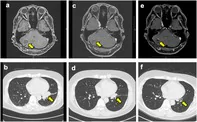

## 1 Introduction

Anaplastic lymphoma kinase (ALK) rearrangements are well‐established oncogenic drivers in non‐small‐cell lung cancer (NSCLC), occurring in approximately 4%–8% of cases [1]. Since the PROFILE 1014 trial demonstrated the superiority of crizotinib over chemotherapy [2], subsequent studies—including ALEX and CROWN—have shown significant improvements in progression‐free and overall survival with ALK tyrosine kinase inhibitors (ALK‐TKIs) [3, 4]. As a result, ALK‐TKIs are now considered standard first‐line therapy for patients with ALK‐positive advanced NSCLC.

While most ALK rearrangements in NSCLC involve echinoderm microtubule‐associated protein‐like 4 (EML4) as the fusion partner [5], the increasing use of next‐generation sequencing (NGS) has led to the identification of rare ALK fusion partners [6]. One such fusion partner is the non‐catalytic scaffold protein striatin (STRN), which has been identified as a fusion partner for ALK. The resulting STRN–ALK fusion protein is believed to contribute to tumourigenesis by activating oncogenic signalling pathways [7]. Although STRN–ALK fusions are rare, a limited number of cases have been reported. Emerging evidence suggests that treatment responses in ALK‐positive NSCLC may vary depending on the fusion partner, leading to uncertainty about the optimal therapeutic approach for rare ALK fusion variants [8].

Lorlatinib, a third‐generation ALK‐TKI designed to overcome resistance mutations and improve central nervous system (CNS) penetration, demonstrated superior systemic and intracranial efficacy compared to crizotinib in the CROWN trial for treatment‐naïve ALK‐positive NSCLC [4]. In the recent 7‐year update, median progression‐free survival remained unreached with lorlatinib versus 9.1 months with crizotinib. Furthermore, no new intracranial progression events occurred after the first 30 months of lorlatinib treatment [9]. However, data on the efficacy of lorlatinib as first‐line therapy in patients with rare ALK fusion variants are limited, and its role in STRN–ALK‐positive NSCLC remains unclear. In this report, we present a case of advanced NSCLC with an STRN–ALK fusion and symptomatic brain metastases that achieved durable disease control with first‐line lorlatinib following stereotactic radiosurgery. This case offers valuable insights into the potential role of lorlatinib in this rare molecular subset.

## 2 Case Report

A 45‐year‐old man who had never smoked presented to our hospital in July 2024 with a history of progressive dizziness and recurrent falls over several weeks. He denied experiencing nausea, vomiting, headache, seizures, or impaired consciousness. Upon neurological examination, he was alert and oriented, with a Glasgow Coma Scale score of 15. Right‐sided dysmetria was noted during finger‐to‐nose and heel‐to‐shin testing. No other focal neurological deficits were observed. His Eastern Cooperative Oncology Group performance status was 1.

**FIGURE 1:**
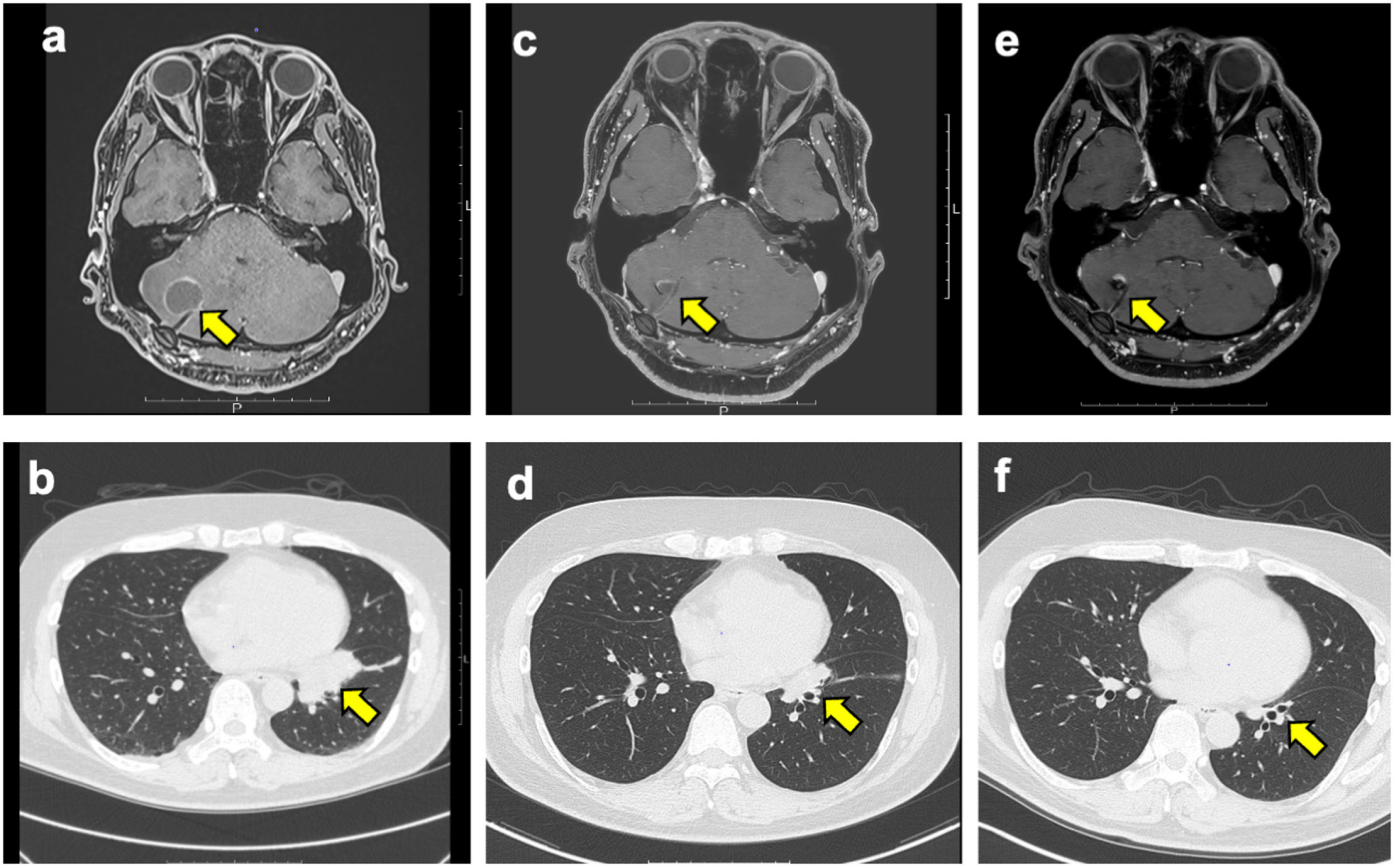
Radiological findings of the present case. (a, c, e) Brain MRI performed before initiation of lorlatinib (a), at 2 months (c), and at 14 months (e), demonstrating marked regression of brain metastases and sustained intracranial disease control. (b, d, f) Representative CT images obtained before lorlatinib initiation (b), at 3 months after treatment initiation (d), and at 17 months during continued therapy (f). Baseline chest CT revealed a tumour lesion in the left lower lobe (b). Follow‐up CT at 3 months showed marked tumour reduction (d), and sustained disease control without progression was maintained at 17 months (f).

Initial laboratory findings were unremarkable, with no evidence of hepatic, renal, or hematologic dysfunction. Tumour markers (CEA 2.1 ng/mL, CA19‐9 28 U/mL, CYFRA 0.6 ng/mL, and Pro‐GRP 22.5 pg/mL) were all within normal limits. Contrast‐enhanced brain magnetic resonance imaging (MRI) revealed a 28 × 24 mm cystic lesion in the right cerebellum (Figure 1a), along with four supratentorial metastatic lesions, each measuring ≤ 30 mm. A chest computed tomography (CT) scan showed a 51 × 22 mm mass in the left lower lobe (Figure 1b), highly suggestive of a primary lung malignancy.

Due to severe dizziness related to posterior fossa involvement, an Ommaya reservoir was placed to aid in intracranial pressure management. Subsequently, all five intracranial metastatic lesions, including the right cerebellar lesion shown in Figure 1a, were treated with stereotactic radiosurgery (35 Gy in five fractions). These interventions led to marked symptomatic improvement. Subsequent bronchoscopic biopsy of the lung lesion confirmed the diagnosis of lung adenocarcinoma. Positron emission tomography–computed tomography revealed metastatic involvement of the left hilar and subcarinal lymph nodes, as well as the left second rib and left iliac bone, consistent with stage IVB disease (T3N2M1c).

Molecular testing was performed using the Oncomine Dx Target Test Multi‐CDx System, an amplicon‐based next‐generation sequencing hotspot panel that analyzes tumour‐derived DNA for hotspot variants in 46 genes and tumour‐derived RNA for fusion transcripts involving 21 genes [10]. This analysis identified an STRN exon 3–ALK exon 20 fusion. Based on this finding, lorlatinib was initiated at a dose of 100 mg once daily as first‐line systemic therapy. Approximately 1 month after starting the treatment, the patient experienced sudden neuropsychiatric adverse events, including mood changes and speech disturbances. These events were classified as Grade 2 according to Common Terminology Criteria for Adverse Events version 5.0, leading to a temporary interruption of lorlatinib for approximately 21 days. The medication was then resumed at a reduced dose of 75 mg. A total of 4 weeks after resumption, recurrent neuropsychiatric symptoms necessitated a further dose reduction to 50 mg. A Grade 1 elevation in low‐density lipoprotein cholesterol was also observed and improved with statin therapy.

Despite the dose reduction, a contrast‐enhanced brain MRI at 2 months showed significant regression of the metastases, with the largest lesion shrinking to 9 × 8 mm (Figure 1c), and all other intracranial lesions remaining stable without progression. Radiological assessment at 3 months revealed a partial response of the primary lung tumour, measuring 23 × 12 mm on chest CT (Figure 1d). A follow‐up MRI at 14 months confirmed sustained intracranial disease control without progression (Figure 1e), while a chest CT at 17 months demonstrated continued tumour shrinkage of the primary lung lesion (Figure 1f). The osseous metastases were assessed using serial non‐contrast CT examinations and showed no apparent radiographic progression. Dedicated PET/CT or bone scintigraphy was not performed. Dose adjustment improved tolerability and allowed for ongoing treatment.

## 3 Discussion

To date, including the present case, 16 cases of STRN–ALK‐positive NSCLC treated with ALK‐TKIs have been reported in the literature (Table 1) [11, 12, 13, 14, 15, 16, 17, 18, 19, 20, 21, 22, 23, 24, 25]. Reported clinical outcomes have been highly heterogeneous, with marked variability in both the magnitude and durability of response. Crizotinib‐containing regimens were used in five cases and were associated with objective reponses, including one complete response and four partial responses, although the durability of response varies. In contrast, second‐generation ALK‐TKIs—most commonly alectinib—demonstrated inconsistent efficacy, with progression‐free survival ranging from only several weeks to more than 1 year [11, 12, 13, 14, 15, 16, 17, 18, 19, 20, 21, 22, 23, 24, 25]. Collectively, these findings suggest that therapeutic sensitivity in STRN–ALK‐positive NSCLC may differ from that observed in tumours harbouring the more common EML4–ALK fusion variants.

**TABLE 1:** Summary of all published case reports of STRN–ALK‐positive NSCLC treated with ALK‐TKIs.

Age	Tissue type	Stage	Brain metastasis	Co‐mutation	Therapy before ALK fusion identification	Therapy after ALK fusion identification	Response	Duration	Causes of PD	Next therapy	Ref No.
59	Adeno	Postoperative recurrence	No	TP53, MYC	No	Crizotinib	CR	6 months ongoing	No		11
51	Adeno	IV	No	Vimentin, ABCB1 mRNA overexpression	Crizotinib	Alectinib	PD	3 months	Pleural effusion, lymph nodes, pericardial fluid	Docetaxel	12
43	Adeno	IVA	No	EGFR ex19 del	Gefitinib	Crizotinib + Gefitinib	PR	6 months ongoing	No	—	13
64	Adeno	IVB	No	SETD2, GRM8	No	Alectinib	PR	19 months ongoing	No	—	14
66	Adeno	IVA	No	MYC	No	Alectinib	PR	6 months ongoing	No	—	15
65	Adeno	IV	No	PIK3CA, KRAS, TP53	No	Alectinib	PD	1.5 months	Enlargement of primary tumour	Crizotinib	16
29	Adeno	IVB	No	PDK1‐ALK, TP53	No	Alectinib	PR	7 months ongoing	No	—	17
38	Not clear	IVA	No	EGFR T790M, EML4‐ALK	Gefitinib	Crizotinib + Osimertinib	PR	—	No	—	18
42	Adeno	Postoperative recurrence	No	No	No	Alectinib	PR	4 months PD	New pulmonary nodules	Crizotinib	19
75	Adeno	IV	No	EGFR ex19 del	Gefitinib	Osimertinib + Alectinib	PD	7 weeks	Iliopsoas	Crizotinib	20
67	Adeno	IVA	No	ALK‐DNAJC27 (A19:D4)	No	Crizotinib	PR	18 months PD	Brain	Ensartinib	21
70	Adeno	III	Not clear	TP53	No	Ensartinib	PR	—	No	—	22
45	Adeno	IVB	No	STK11‐LKB1	No	Alectinib	PR	6 months PD	Brain	Lorlatinib	23
52	Adeno	IVA	Not clear	Not reported	No	Crizotinib	PR	4 years	Brain, anterior left chest metastasis	Ceritinib	24
52	Adeno	IVB	No	ARID1A, KMT2D	No	Lorlatinib	CR	9 months	No		25
45	Adeno	IVB	Yes	No	Stereotactic radiosurgery	Lorlatinib	PR	17 months ongoing	No	—	Present case

Abbreviations: Adeno; adenocarcinoma, CR; complete response, PD; progressive disease, PR; partial response.

CNS involvement represents a major clinical challenge in ALK‐positive NSCLC. As summarized in Table 1, several patients with STRN–ALK‐positive disease developed brain metastases either at diagnosis or during the disease course [11, 12, 13, 14, 15, 16, 17, 18, 19, 20, 21, 22, 23, 24, 25]. Given that up to 40% of patients with ALK‐positive NSCLC ultimately develop CNS metastases, achieving durable intracranial disease control remains a critical therapeutic objective [26, 27]. The relatively high frequency of CNS involvement among reported STRN–ALK‐positive cases further underscores the importance of selecting ALK‐TKIs with strong CNS activity in this rare molecular subset.

Lorlatinib is a third‐generation ALK‐TKI specifically engineered to overcome resistance mutations and to achieve enhanced CNS penetration. In treatment‐naïve ALK‐positive NSCLC, lorlatinib demonstrated superior systemic and intracranial efficacy compared with crizotinib in the CROWN trial, with durable benefit confirmed in the recent 7‐year update [4, 9]. However, ALK positivity in the CROWN trial was determined using the Ventana ALK (D5F3) CDx immunohistochemistry assay, which does not identify specific ALK fusion partners or variants. Accordingly, outcomes specifically for patients with STRN–ALK fusions were not reported, and the applicability of the CROWN results to this rare molecular subgroup remains uncertain. Among previously reported STRN–ALK‐positive cases, only one patient received lorlatinib as second‐line therapy following alectinib failure and derived limited clinical benefit [23]. Based on that observation, it was hypothesized that structural features of the STRN–ALK fusion protein might reduce sensitivity to certain ALK‐TKIs, potentially contributing to intrinsic resistance.

This case adds to the limited reports of first‐line lorlatinib use in STRN–ALK‐positive NSCLC. Durable systemic and intracranial disease control was achieved despite the need for stepwise dose reductions due to neuropsychiatric adverse events. Notably, tumour regression was maintained at reduced doses, and disease control persisted for more than 14 months. These findings indicate that lorlatinib may retain clinically meaningful antitumor activity in selected patients with STRN–ALK‐positive disease, particularly when used early in the treatment course.

Heterogeneity in treatment response among patients with STRN–ALK‐positive NSCLC is likely influenced not only by the fusion partner itself but also by coexisting resistance mechanisms [28, 29]. In the limited number of published cases summarized in Table 1, short‐lived responses to ALK‐TKIs were frequently associated with concomitant genomic alterations implicated in ALK‐independent resistance, including *ABCB1* overexpression, *STK11*/*LKB1* mutations, and *PIK3CA* mutations [12, 16, 23]. Similar ALK‐independent resistance mechanisms have been described more broadly in ALK‐rearranged NSCLC [28, 29, 30, 31, 32]. In a previously reported case treated with first‐line lorlatinib, pathogenic co‐alterations in ARID1A and KMT2D were identified; however, these alterations are not currently established mechanisms of ALK‐TKI resistance. In both the previously reported and the present cases, no known ALK‐independent resistance‐associated co‐mutations were identified, which may partly account for the favourable response to lorlatinib.

Emerging evidence further suggests that STRN–ALK and other non‐EML4 ALK fusion variants may be enriched in the setting of secondary ALK rearrangements. Although EGFR and ALK alterations are generally considered mutually exclusive [33], concomitant alterations have been reported [34] and secondary acquisition of ALK rearrangements during EGFR‐TKI therapy has been documented in a small subset of EGFR‐mutant NSCLC [13, 18, 20, 35]. In these cases, non‐EML4 fusion partners accounted for approximately one‐third of ALK rearrangements [35], a substantially higher proportion than that observed in *de novo* ALK‐positive NSCLC, in which EML4–ALK predominates [5]. These findings suggest that secondary ALK fusions, including STRN–ALK, may arise through distinct biological mechanisms compared with primary ALK rearrangements, potentially contributing to differences in therapeutic sensitivity.

Several limitations of this report should be acknowledged. First, molecular profiling was performed using a limited gene panel, and additional genomic alterations may therefore have gone undetected. Second, stereotactic radiosurgery was administered prior to initiation of lorlatinib, which may have contributed to intracranial disease control and confounded assessment of lorlatinib's isolated CNS efficacy. Finally, as a single case report, generalizability is limited.

A previous report has suggested the potential efficacy of lorlatinib as first‐line therapy in STRN–ALK‐positive NSCLC [25]. In the present case, durable disease control was achieved despite multiple brain metastases at diagnosis, with subsequent local radiotherapy. Furthermore, antitumor activity was maintained even after dose reduction due to adverse events. These findings extend the current evidence and suggest that lorlatinib may provide sustained systemic and intracranial control with manageable toxicity in selected patients.

## Author Contributions

Masaru Maebeya and Shinobu Tamura conceptualized the idea of writing a case report and wrote the manuscript with support from Takeshi Shono, Takanobu Hoso, and Hitomi Tatsuta. All authors contributed to diagnosis and management. Shinobu Tamura made critical revisions to the manuscript. All authors have read and approved the final manuscript.

## Funding

This work was supported by the JSPS KAKENHI Grant Number (JP23K07865).

## Consent

The authors declare that written informed consent was obtained for the publication of this manuscript and accompanying images using the consent form provided by the Journal.

## Conflicts of Interest

The authors declare no conflicts of interest.

## Data Availability

The data that support the findings of this study are available on request from the corresponding author. The data are not publicly available due to privacy or ethical restrictions.
